# Protective Effect of Ergothioneine against Oxidative Stress-Induced Chondrocyte Death

**DOI:** 10.3390/antiox13070800

**Published:** 2024-07-01

**Authors:** Shuzo Sakata, Ryo Kunimatsu, Kotaro Tanimoto

**Affiliations:** 1 Department of Orthodontics, Division of Oral Health and Development, Hiroshima University Hospital, 1-2-3 Kasumi, Minami-ku, Hiroshima 734-8553, Japan; shuzosakata@hiroshima-u.ac.jp; 2 Department of Orthodontics and Craniofacial Developmental Biology, Graduate School of Biomedical and Health Sciences, Hiroshima University, 1-2-3 Kasumi, Minami-ku, Hiroshima 734-8553, Japan; tkotaro@hiroshima-u.ac.jp

**Keywords:** reactive oxygen species, ergothioneine, chondrocyte protection, rheumatoid arthritis, oxidative stress

## Abstract

Reactive oxygen species (ROS) induce oxidative stress in cells and are associated with various diseases, including autoimmune diseases. Ergothioneine (EGT) is a natural amino acid derivative derived from the ergot fungus and has been reported to exhibit an effective antioxidant function in many models of oxidative stress-related diseases. Recently, mutations in OCTN1, a membrane transporter of EGT, have been reported to be associated with rheumatoid arthritis. Therefore, we investigated the chondrocyte-protective function of EGT using a model of oxidative stress-induced injury of chondrocytes by hydrogen peroxide (H_2_O_2_). Human chondrocytes were subjected to oxidative stress induced by H_2_O_2_ treatment, and cell viability, the activity of lactate dehydrogenase (LDH) released into the medium, dead cell ratio, intracellular ROS production, and mitochondrial morphology were assessed. EGT improved chondrocyte viability and LDH activity in the medium and strongly suppressed the dead cell ratio. EGT also exerted protective effects on intracellular ROS production and mitochondrial morphology. These results provide evidence to support the protective effects of EGT on chondrocytes induced by oxidative stress.

## 1. Introduction

Reactive oxygen species (ROS) are a group of highly oxidatively active molecules that are produced during oxidative phosphorylation in mitochondria, and ROS are associated with the various physiological functions of cells [1]. ROS can be produced by many factors such as ultraviolet (UV) light, radiation, smoking, aging, food, and inflammation. Cells have ROS scavenging systems such as superoxide dismutase, catalase, and glutathione peroxidase, which maintain the homeostasis of intracellular ROS concentrations. However, when the production of ROS exceeds the antioxidant capacity of the cell and the homeostasis of intracellular ROS concentration is disrupted, intracellular lipids, enzymes, and nucleic acids are peroxidized, disrupting the maintenance of vital functions [2]. This condition is called oxidative stress, and oxidative stress caused by the excessive accumulation of ROS has been reported to be involved in many diseases, including cancer [3], hypertension [4], atherosclerosis [5], neurodegenerative diseases [6], diabetes [7], and autoimmune diseases [8].

Recently, oxidative stress has been reported to be involved in rheumatoid arthritis (RA) and osteoarthritis (OA) [9,10]. These diseases are caused by the chronic inflammation of the synovial membrane, which leads to the degeneration of bone, cartilage, and other joint tissues [11]. The excessive accumulation of ROS in chondrocytes induces mitochondrial dysfunction associated with membrane lipid peroxidation, leading to chondrocyte death [12]. Since articular cartilage has low proliferative capacity and lacks the ability to self-repair, chondrocyte death is considered an important factor in the pathological progression of joint-destructive diseases [13]. Therefore, the reduction of excess intracellular ROS accumulation and the protection of cellular physiological functions by supplementation with antioxidants could be an effective therapeutic strategy against oxidative stress-related diseases. The standard treatment for OA and RA is the oral administration of anti-rheumatic drugs, non-steroidal anti-inflammatory drugs, and steroids, or the intra-articular injection of hyaluronic acids; however, these treatments have certain side effects [14,15]. Therefore, the development of a safe supplement therapy that complements the patient’s antioxidant capacity through the intake of dietary antioxidants is necessary [16].

Ergothioneine (EGT) is a unique amino acid derivative derived from the ergot fungus and has a strong antioxidant capacity and high chemical stability [17]. The potential efficacy of EGT as a therapeutic agent is becoming clear, with many reports that EGT is effective in neurodegenerative diseases such as Parkinson’s disease caused by neuronal oxidative stress [18,19]. Recently, OCTN1, a plasma membrane transporter of EGT, has been reported to be associated with RA [20]. These reports suggest that investigating the effects of EGT on oxidative stress in chondrocytes is important for establishing new therapeutic strategies for OA and RA. Therefore, this study aimed to examine the potential effectiveness of EGT for degenerative diseases of articular cartilage by evaluating the protective effect of EGT against the cytotoxicity induced by oxidative stress.

## 2. Materials and Methods

### 2.1. Cell Culture

Primary normal human knee articular chondrocytes (NHAC-Kn) were obtained from Lonza (Walkersville, MD, USA). Chondrocytes were cultured in chondrocyte basal medium (Lonza) supplemented with 5% fetal bovine serum (FBS), 0.2% R3-insulin-like-growth factor-1, 0.5% human recombinant fibroblast growth factor-beta, 1% transferrin, 0.1% insulin, and 0.1% gentamicin/amphotericin-B according to the manufacturer’s instructions. NHAC-Kn cells were maintained in a 37 °C humidified atmosphere of 5% CO_2_ and used for all experiments at passage 5.

### 2.2. Quantitative Polymerase Chain Reaction (qPCR)

Total RNA was extracted from chondrocytes treated with 1 mM EGT for 24 h using an RNeasy Mini Kit (Qiagen, Valencia, CA, USA) according to the manufacturer’s instructions. The control group included chondrocytes treated with phosphate-buffered saline (PBS), the vehicle for EGT. Complementary DNA (cDNA) was synthesized from 1 µg of total RNA using a ReverTra Ace qPCR Master Mix (Toyobo, Osaka, Japan) and SimpliAmp Thermal Cycler (Thermo Fisher Scientific, Waltham, MA, USA). qPCR was performed using a Realtime PCR Master Mix (Toyobo) and a Thermal Cycler Dice Real Time System (Takara, Shiga, Japan). The following primers were used for qPCR: *collagen type 2 alpha 1 (COL2A1)*, forward 5′-GCTCCCAGAACATCACCTACCA-3′, reverse 5′-AACAGTCTTGCCCCACTTACCG-3′ and actin beta (*ACTB*); forward 5′-AGAGCTACGAGCTGCCTGAC-3′ and reverse 5′-AGCACTGTGTTGGCGTACAG-3′. *COL2A1* mRNA levels were normalized to *ACTB* mRNA levels, and the relative gene expression of *COL2A1* was calculated with the 2^−ΔΔCT^ method.

### 2.3. Cell Viability and Cytotoxicity Analysis

Cell viability was examined using a Cell Counting Kit-8 (CCK-8, Dojindo, Kumamoto, Japan). The chondrocytes were seeded with 5000 cells in a 96-well plate, and after 24 h incubation, the cells were treated with EGT (0, 0.1, 0.25, 0.5, and 1 mM) or hydrogen peroxide (H_2_O_2_; 0, 0.1, 0.25, 0.5, and 1 mM). After 24 h incubation, CCK-8 solution was added to the medium for 1 h at 37 °C. The absorbance at 450 nm was measured by using a microplate reader Multiskan FC (Themo scientific, Waltham, MA, USA).

Cell cytotoxicity was examined using a Cytotoxicity LDH Assay Kit-WST (Dojindo). Chondrocytes were seeded with 10,000 cells in a 24-well plate, and after 24 h incubation, the cells were treated with H_2_O_2_ (1 mM) or with the addition of EGT (0.1, 0.25, 0.5, and 1 mM). After an additional 24 h incubation, the activity of lactate dehydrogenase (LDH) in the supernatant was measured following the manufacturer’s instructions. Cell morphology and confluency were analyzed by using CELLCYTE X (CYTENA, Freiburg, Germany).

### 2.4. Live/Dead-Cell Assay

To detect H_2_O_2_-induced chondrocyte death, Diyo-1 (AAT Bioquest, Sunnyvale, CA, USA) and SYTO-59 (Invitrogen, Carlsbad, CA, USA) double-staining was performed. Diyo-1 is a membrane-impermeable nuclear-staining dye that penetrates dead cell membranes and emits green fluorescence. By contrast, SYTO-59 is a membrane-permeable nuclear-staining dye that can penetrate the cell membrane of both dead and living cells and emits red fluorescence. Therefore, the ratio of dead cells to total cells can be calculated by dividing the number of green fluorescence-stained cells by the number of red fluorescence-stained cells. These fluorescent dyes were dissolved at a concentration of 1 µM each in chondrocyte basal medium containing FBS and supplements. Chondrocytes were seeded with 10,000 cells in a 24-well plate, and after 24 h incubation, the cells were treated with H_2_O_2_ (1 mM) or with the addition of EGT (1 mM) at 37 °C for 3, 6, and 12 h. The control group was treated with PBS. Following treatments, fluorescence images were obtained by using CELLCYTE X (CYTENA).

### 2.5. ROS Assay

Intracellular ROS production was evaluated using dichloro-dihydro-fluorescein diacetate (DCFH-DA) dye (ROS Assay Kit Photo-oxidation Resistant DCFH-DA, Dojindo). The chondrocytes were treated with H_2_O_2_ (1 mM) and EGT (1 mM) at 37 °C for 3, 6, and 12 h in the presence of DCFH-DA dye according to the manufacturer’s instructions. The control group was treated with PBS. Live-cell fluorescence images were obtained and analyzed by using CELLCYTE X (CYTENA).

### 2.6. Mitochondrial Staining

Mitochondrial visualization was performed using MitoView Green (Biotium, San Francisco, CA, USA). This probe is independent of mitochondrial membrane potential and fluoresces when fractionated into the mitochondrial membrane. Therefore, mitochondrial mass can be evaluated. Chondrocytes were treated with H_2_O_2_ (1 mM) and EGT (1 mM) at 37 °C for 2 h in the presence of MitoView Green (200 nM), and fluorescence images were obtained using CELLCYTE X (CYTENA).

### 2.7. Statistical Analysis

The results are shown as mean ± standard deviation (SD). Statistical analysis was performed using JMP Pro version 17 software (JMP Statistical Discovery LlC, Cary, NC, USA) and statistically significant differences between the groups were determined using one-way analysis of variance followed by Dunnett’s test or Tukey’s test. The statistical significance was set at *p* < 0.05.

## 3. Results

### 3.1. Effect of EGT and H_2_O_2_ on Collagen Gene Expression and Cell Viability

**Figure 1:**
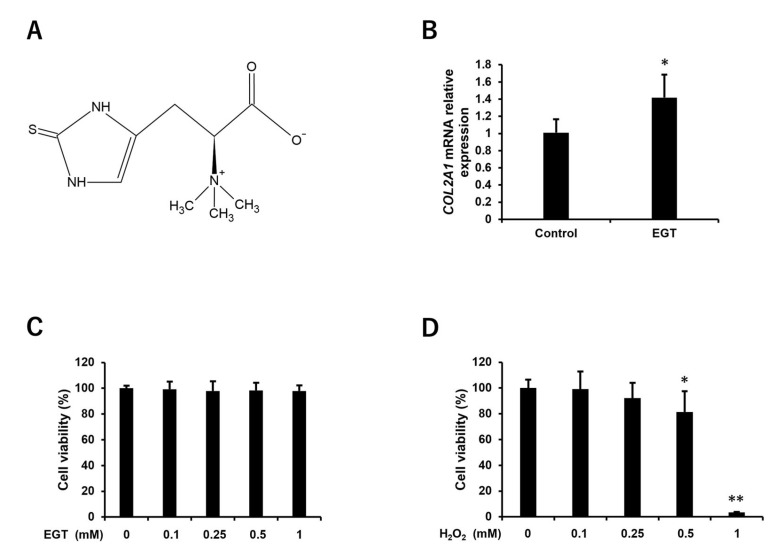
Effect of EGT and H_2_O_2_ on cell viability of human chondrocytes. (**A**) Structural formula of EGT. (**B**) Relative gene expression of *COL2A1* in chondrocytes treated with EGT (1 mM) for 24 h. *COL2A1* mRNA levels were normalized to ACTB mRNA levels. The values are presented as mean ± SD (n = 4). (**C**) Cell viability of chondrocytes treated with EGT at various concentrations (0–1 mM) for 24 h. (**D**) Cell viability of chondrocytes treated with H_2_O_2_ at various concentrations (0–1 mM) for 24 h. Cell viability was determined by CCK-8 assay. The values of cell viability are presented as mean ± SD (n = 8). * *p* < 0.05, ** *p* < 0.01 vs. control group. EGT, ergothioneine; H_2_O_2_, hydrogen peroxide; *COL2A1*, collagen type 2 alpha 1; ACTB, actin beta; CCK-8, Cell Counting Kit-8; SD, standard deviation.

An increase in the gene expression of COL2A1 in chondrocytes treated with EGT was observed compared to untreated chondrocytes (Figure 1B). When treating the chondrocytes with 0.1, 0.25, 0.5, and 1 mM of EGT for 24 h, no significant difference was observed (Figure 1C). On the other hand, on application of 0.1, 0.25, 0.5, and 1 mM of H_2_O_2_ to chondrocytes for 24 h, a significant decrease in cell viability of approximately 20% was observed at 0.5 mM H_2_O_2_, and a significant decrease of approximately 95% was observed at 1 mM (Figure 1D).

### 3.2. EGT Ameliorates H_2_O_2_-Induced Chondrocyte Damage

**Figure 2:**
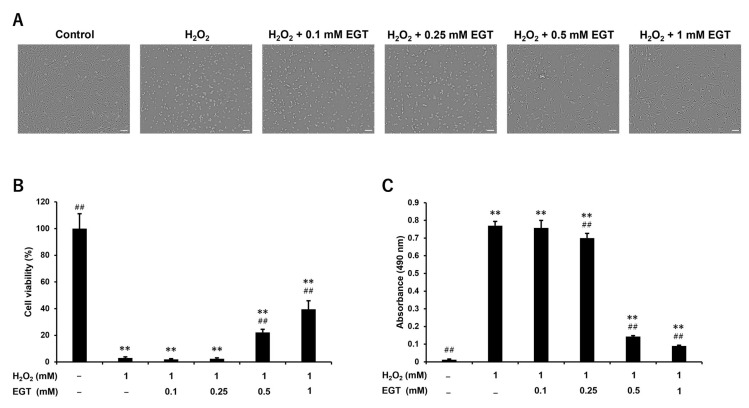
EGT ameliorates H_2_O_2_-induced cell damage in chondrocytes. (**A**) Morphology of chondrocytes treated with H_2_O_2_ (1 mM) and EGT (0–1 mM) for 24 h (scale bar: 100 µm). (**B**) Cell viability quantified by CCK-8 assay. Values are presented as mean ± SD (n = 6). (**C**) LDH activity of supernatant of chondrocytes treated with H_2_O_2_ (1 mM) and EGT (0–1 mM) for 24 h. LDH activity was measured using Cytotoxicity LDH Assay Kit-WST. Values are presented as mean ± SD (n = 6). ** *p* < 0.01 vs. control group; ## *p* < 0.01 vs. H_2_O_2_-treated group. EGT, ergothioneine; H_2_O_2_, hydrogen peroxide; CCK-8, Cell Counting Kit-8; LDH, lactate dehydrogenase; SD, standard deviation.

In comparison, when treating the chondrocytes with 0.1, 0.25, 0.5, and 1 mM of EGT in addition to 1 mM H_2_O_2_, the addition of 0.5 and 1 mM of EGT improved the morphological changes in chondrocytes and decreased the viability caused by H_2_O_2_ (Figure 2A,B). Similarly, the activity of extracellularly released LDH, which was increased by H_2_O_2_ treatment, was significantly decreased by the addition of 0.5 and 1 mM of EGT (Figure 2C).

### 3.3. Inhibitory Effect of EGT on H_2_O_2_-Induced Chondrocyte Death

**Figure 3:**
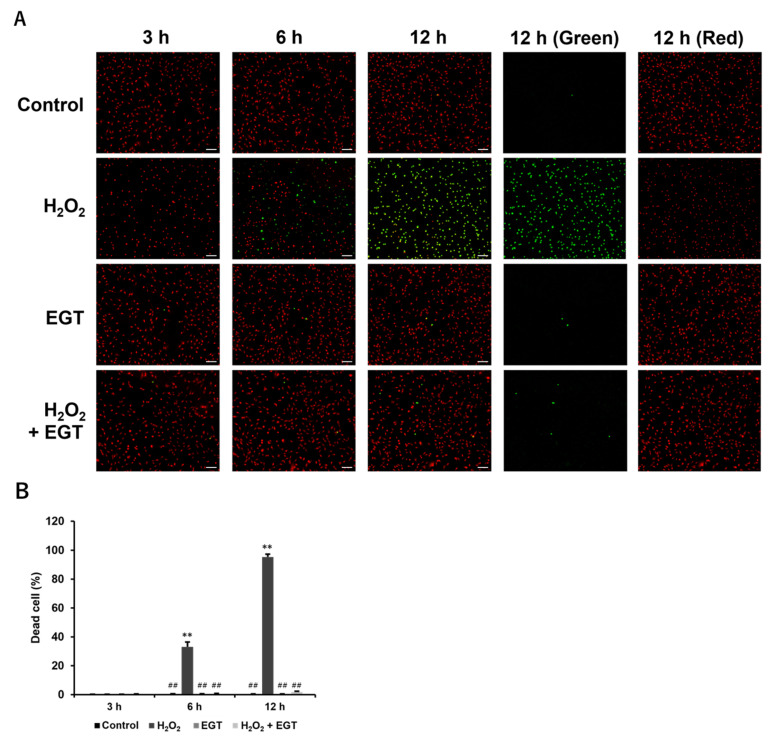
EGT inhibits H_2_O_2_-induced cell death in chondrocytes. (**A**) Fluorescence image of chondrocytes treated with H_2_O_2_ (1 mM) and EGT (1 mM) for 3, 6, and 12 h (scale bar: 100 µm). Dead cells (green) and total cells (red) were double-stained with Diyo-1 and SYTO59 nuclear staining dyes. (**B**) Quantitative data on the ratio of dead cells to total cells calculated by double-staining. Values are presented as mean ± SD (n = 3). ** *p* < 0.01 vs. control group; ## *p* < 0.01 vs. H_2_O_2_-treated group. EGT, ergothioneine; H_2_O_2_, hydrogen peroxide; SD, standard deviation.

The essential mechanism of cell death by treatment with H_2_O_2_ is the peroxidation of polyunsaturated fatty acids by excess intracellular ROS accumulation and subsequent disruption of cell membrane continuity [21,22]. Therefore, the double-staining assay for live and dead cells using the difference in cell membrane permeability of nuclear-staining dyes was performed. No dead cells were observed at 3 h after the addition of 1 mM H_2_O_2_ and 1 mM EGT, but a significant increase in the dead cell ratio was observed in the H_2_O_2_-treated group at 6 h after treatment (Figure 3A,B). After 12 h of treatment, most of the cells were identified as dead cells in the H_2_O_2_-treated group. On the other hand, in the H_2_O_2_ + EGT-treated group, these cell deaths were strongly inhibited and no significant increase in the dead cell ratio was observed at 12 h after treatment. No significant change in the dead cell ratio was observed in the group treated with EGT compared to the control group.

### 3.4. Effect of EGT on ROS Production in H_2_O_2_-Treated Chondrocytes

**Figure 4:**
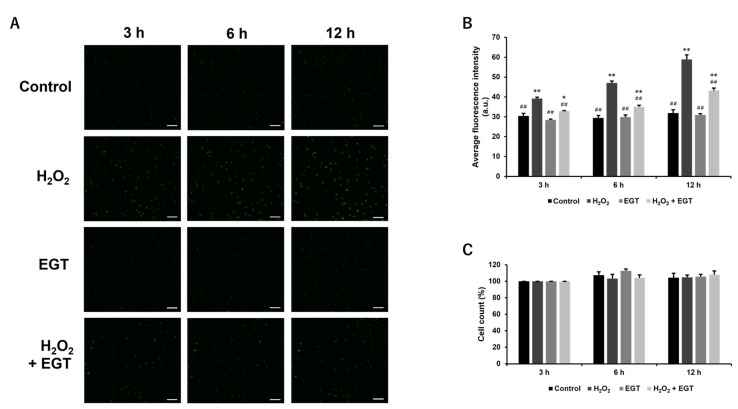
EGT inhibits H_2_O_2_-induced intracellular ROS production in chondrocytes. (**A**) Fluorescence images of chondrocytes treated with H_2_O_2_ (1 mM) and EGT (1 mM) for 3, 6, and 12 h in the presence of DCFH-DA dye (scale bar: 100 µm). (**B**) Quantitative data on the average fluorescence intensity of chondrocytes obtained from fluorescence images. Values are presented as mean ± SD (n = 3). (**C**) Changes in cell count over time. The values are expressed as 100% of the cell count in each group at 3 h. * *p* < 0.05, ** *p* < 0.01 vs. control group; ## *p* < 0.01 vs. H_2_O_2_-treated group. EGT, ergothioneine; H_2_O_2_, hydrogen peroxide; ROS, reactive oxygen species; SD, standard deviation; DCFH-DA, dichloro-dihydro-fluorescein diacetate.

Cells treated with H_2_O_2_ have increased intracellular ROS concentrations. Therefore, intracellular ROS accumulation was monitored using DCFH-DA dye, a fluorescent probe for intracellular ROS. Consequently, significant increases in intracellular ROS concentrations were observed at 3, 6, and 12 h after H_2_O_2_ treatment, but the addition of EGT significantly suppressed these increases (Figure 4A,B). No significant changes in intracellular ROS concentrations were observed after treatment with EGT. No changes in cell counts were observed (Figure 4C).

### 3.5. Effect of EGT on Mitochondrial Morphology of H_2_O_2_-Treated Chondrocytes

**Figure 5:**
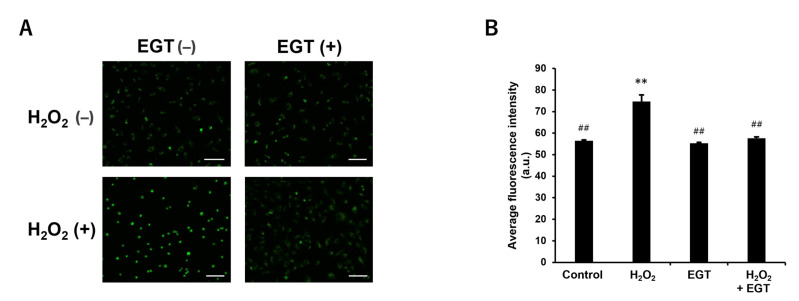
EGT inhibits H_2_O_2_-induced mitochondrial condensation in chondrocytes. (**A**) Fluorescence images of chondrocytes treated with H_2_O_2_ (1 mM) and EGT (1 mM) for 2 h in the presence of MitoView Green dye (scale bar: 100 µm). (**B**) Quantitative data on the average fluorescence intensity of chondrocytes obtained from fluorescence images. Values are presented as mean ± SD (n = 3). ** *p* < 0.01 vs. control group; ## *p* < 0.01 vs. H_2_O_2_-treated group. EGT, ergothioneine; H_2_O_2_, hydrogen peroxide; SD, standard deviation.

Cell death due to oxidative stress causes mitochondrial dysfunction in its early stages [23]. Therefore, mitochondrial staining using MitoView Green dye was performed to observe mitochondrial morphology in the pre-membrane disruption phase caused by H_2_O_2_. In chondrocytes treated with H_2_O_2_ for 2 h, mitochondrial condensation was observed prior to plasma membrane damage, with a significant increase in fluorescence intensity (Figure 5A,B). However, no significant change in fluorescence intensity was observed in the H_2_O_2_ + EGT- and EGT-treated groups compared to the control group.

## 4. Discussion

EGT is a naturally occurring amino acid derivative with safe and potent antioxidant properties that may be a potential therapeutic agent for oxidative stress-related diseases. In this study, to examine the chondrocyte-protective effects of EGT, an oxidative stress-induced chondrocyte injury model was generated using H_2_O_2_. The results showed that 1 mM EGT potently inhibited H_2_O_2_-induced chondrocyte death, demonstrating its efficacy in a model of oxidative stress-induced cartilage damage in vitro.

Firstly, the cytotoxicity of EGT on chondrocytes was investigated. The results of the CCK-8 assay showed that EGT has no cytotoxicity to chondrocytes up to a concentration of 1 mM. Previous studies have reported no cytotoxicity after 24 h of exposure to 1 mM EGT [24,25,26], which is consistent with our results. However, some reports have suggested that even concentrations not reaching 1 mM may be cytotoxic [27,28], possibly due to differences in the expression of the EGT transporter (OCTN1) in different cell types. OCTN1 has been found to be widely distributed in many mammalian organs and cells [29]. Accordingly, EGT, the substrate of OCTN1, is absorbed from the small intestine and then transported to all tissues in the body and retained for a long period of time [30]. Therefore, when administrating EGT to the human body, the appropriate EGT-application concentration should be carefully considered.

The protective effect of EGT on chondrocytes against H_2_O_2_-induced oxidative stress was evaluated using the LDH-release assay and the cell nuclear double-staining assay. In this study, chondrocyte damage caused by 1 mM H_2_O_2_ was inhibited in a dose-dependent manner by the addition of EGT. In addition, 1 mM EGT markedly inhibited chondrocyte death.

H_2_O_2_ is most commonly used as an oxidant for oxidative stress-induced cellular damage [31,32,33,34]. Several studies have reported on the protective effects of EGT against H_2_O_2_-induced oxidative stress, including reports on phaeochromocytoma [35] and hippocampal neural cells [36]. Similar to these studies, the results of this study showed that EGT had a cytoprotective effect against H_2_O_2_-induced oxidative stress.

On monitoring intracellular ROS using a fluorescence probe, EGT significantly inhibited the H_2_O_2_-induced increase in intracellular ROS concentration. This is similar to previous studies [37]. EGT did not reduce intracellular ROS concentration exclusively.

H_2_O_2_, a metabolite of superoxide, is produced mainly by leukocytes and macrophages and has harmful effects on normal tissue in autoimmune diseases such as RA [38]. Under physiological conditions, the extracellular concentration of H_2_O_2_, the most stable ROS, is up to approximately 10 µM, whereas under pathological conditions, the concentration of ROS reaches as high as 1 mM, nearly 100 times higher than that in natural conditions [39,40].

ROS are not only harmful to the organism but are also involved in physiological activities [41]. EGT reacts strongly with free radicals, including hydroxyl radicals, whereas it is less directly reactive with H_2_O_2_ [42,43]. This property implies that only the most dangerous hydroxyl radicals can be scavenged without excessively scavenging the ROS necessary for physiological activity. In the present study, EGT treatment alone was able to potently inhibit cell death without showing cytotoxicity, which may be due to this property. Further, this property may be responsible for EGT not reducing the intracellular ROS production exclusively.

Finally, mitochondrial morphology was observed using MitoView Green. Hydroxyl radicals produced in mitochondria peroxidize mitochondrial membranes, leading to mitochondrial dysfunction [44,45]. In the present study, EGT suppressed the changes in mitochondrial morphology caused by H_2_O_2_. This indicates that EGT protects mitochondria from oxidative stress damage caused by H_2_O_2_.

Several studies have tested the efficacy of antioxidant therapy for RA [46,47,48]. EGT, similar to these antioxidants, is possibly safer because it does not violate the body’s natural ROS homeostasis, which is expected to be effective against RA. Further, drug therapy for RA may elevate oxidative stress in patients [49,50]. EGT is also expected to serve as an adjunctive supplement to reduce these side effects of conventional drug therapy.

The present study investigated H_2_O_2_-induced chondrocyte death, and the findings are consistent with previous findings conducted with the naturally occurring antioxidants vitamin C and vitamin E [51,52]. Compared to these antioxidants, however, EGT has pharmacokinetic properties more suitable for supplement therapy as its blood levels are maintained for long periods of time after a single administration. Hence, EGT could be a better antioxidant supplement.

However, the future application of antioxidant therapy with EGT for RA requires the further evaluation of the efficacy and safety of EGT in vivo. In addition, since EGT is transported to organs throughout the body via the ubiquitously expressed OCTN1, its systemic effects must also be carefully investigated.

This study has a few limitations. First, the results of this study did not directly demonstrate efficacy against RA. Further studies in disease models using rheumatoid cells from patients are needed to further evaluate efficacy. Furthermore, the pathogenesis of RA is complex, and, in addition to oxidative stress, many factors contribute, including chronic inflammatory conditions and cartilage matrix destruction. Therefore, further detailed studies using cellular and animal models of inflammation and matrix destruction are needed to explore the potential therapeutic effects of EGT for RA.

## 5. Conclusions

In this study, EGT potently inhibited chondrocyte death induced by H_2_O_2_ without harming chondrocytes. EGT also improved ROS production and the mitochondrial morphological changes caused by H_2_O_2_. This result suggests the efficacy of EGT in oxidative stress-induced chondrocyte damage. Thus, EGT may be a candidate therapeutic agent for degenerative diseases caused by oxidative stress.

## Data Availability

The data supporting the findings of this study are available from the corresponding author upon reasonable request.
